# Toward Measuring *Schistosoma* Response to Praziquantel Treatment with Appropriate Descriptors of Egg Excretion

**DOI:** 10.1371/journal.pntd.0003821

**Published:** 2015-06-18

**Authors:** Piero L. Olliaro, Michel Vaillant, Aïssatou Diawara, Jean T. Coulibaly, Amadou Garba, Jennifer Keiser, Charles H. King, Stefanie Knopp, Aly Landouré, Eliézer K. N’Goran, Giovanna Raso, Alexandra U. Scherrer, José Carlos Sousa-Figueiredo, Katarina Stete, Xiao-Nong Zhou, Jürg Utzinger

**Affiliations:** 1 UNICEF/UNDP/World Bank/WHO Special Programme on Research and Training in Tropical Diseases (TDR), World Health Organization, Geneva, Switzerland; 2 Centre for Tropical Medicine, Nuffield Department of Medicine, University of Oxford, Oxford, United Kingdom; 3 Centre of Competence for Methodology and Statistics (CCMS), Luxembourg Institute of Health (LIH), Strassen, Luxembourg; 4 Department of Biology, Division of Science and Mathematics, New York University Abu Dhabi, Abu Dhabi, United Arab Emirates; 5 Unité de Formation et de Recherche Biosciences, Université Félix Houphouët-Boigny, Abidjan, Côte d’Ivoire; 6 Centre Suisse de Recherches Scientifiques en Côte d’Ivoire, Abidjan, Côte d’Ivoire; 7 Department of Medical Parasitology and Infection Biology, Swiss Tropical and Public Health Institute, Basel, Switzerland; 8 University of Basel, Basel, Switzerland; 9 Department of Epidemiology and Public Health, Swiss Tropical and Public Health Institute, Basel, Switzerland; 10 Réseau International Schistosomoses, Environnement, Aménagement et Lutte (RISEAL-Niger), Niamey, Niger; 11 Center for Global Health and Diseases, Case Western Reserve University, Cleveland, Ohio, United States of America; 12 Schistosomiasis Consortium for Operational Research and Evaluation, University of Georgia, Athens, Georgia, United States of America; 13 Wolfson Wellcome Biomedical Laboratories, Department of Life Sciences, Natural History Museum, London, United Kingdom; 14 Institut National de Recherche en Santé Publique, Bamako, Mali; 15 Division of Infectious Diseases and Hospital Epidemiology, University Hospital Zurich, University of Zurich, Zurich, Switzerland; 16 Department of Infectious and Tropical Diseases, London School of Hygiene and Tropical Medicine, London, United Kingdom; 17 Center for Infectious Diseases and Travel Medicine, Department of Medicine, University Hospital Freiburg, Freiburg im Breisgau, Germany; 18 National Institute of Parasitic Diseases, Chinese Center for Disease Control and Prevention, Shanghai, People’s Republic of China; 19 Key Laboratory of Parasite and Vector Biology of the Chinese Ministry of Health, WHO Collaborating Center for Malaria, Schistosomiasis and Filariasis, Shanghai, People’s Republic of China; Queensland Institute for Medical Research, AUSTRALIA

## Abstract

**Background:**

The control of schistosomiasis emphasizes preventive chemotherapy with praziquantel, which aims at decreasing infection intensity and thus morbidity in individuals, as well as transmission in communities. Standardizing methods to assess treatment efficacy is important to compare trial outcomes across settings, and to monitor program effectiveness consistently. We compared customary methods and looked at possible complementary approaches in order to derive suggestions for standardizing outcome measures.

**Methodology/Principal Findings:**

We analyzed data from 24 studies conducted at African, Asian, and Latin American sites, enrolling overall 4,740 individuals infected with *Schistosoma mansoni*, *S*. *haematobium*, or *S*. *japonicum*, and treated with praziquantel at doses of 40–80 mg/kg. We found that group-based arithmetic and geometric means can be used interchangeably to express egg reduction rates (ERR) only if treatment efficacy is high (>95%). For lower levels of efficacy, ERR estimates are higher with geometric than arithmetic means. Using the distribution of individual responses in egg excretion, 6.3%, 1.7% and 4.3% of the subjects treated for *S*. *haematobium*, *S*. *japonicum* and *S*. *mansoni* infection, respectively, had no reduction in their egg counts (ERR = 0). The 5^th^, 10^th^, and 25^th^ centiles of the subjects treated for *S*. *haematobium* had individual ERRs of 0%, 49.3%, and 96.5%; the corresponding values for *S*. *japonicum* were 75%, 99%, and 99%; and for *S*. *mansoni* 18.2%, 65.3%, and 99.8%. Using a single rather than quadruplicate Kato-Katz thick smear excluded 19% of *S*. *mansoni*-infected individuals. Whilst the effect on estimating ERR was negligible by individual studies, ERR estimates by arithmetic means were 8% lower with a single measurement.

**Conclusions/Significance:**

Arithmetic mean calculations of *Schistosoma* ERR are more sensitive and therefore more appropriate to monitor drug performance than geometric means. However, neither are satisfactory to identify poor responders. Group-based response estimated by arithmetic mean and the distribution of individual ERRs are correlated, but the latter appears to be more apt to detect the presence and to quantitate the magnitude of suboptimal responses to praziquantel.

## Author Summary

To identify whether a person is infected with parasitic worms, stool or urine samples are examined for worm eggs. The drug praziquantel is used against the parasitic disease schistosomiasis. However, there is no definitive agreement as to how the efficacy of praziquantel is best expressed. We put together a database from various studies of the efficacy of praziquantel against schistosomiasis. Efficacy was measured using customary methods: cure rate (CR: percentage of people with eggs in their stool/urine before treatment who became egg-negative after treatment); and egg reduction rate (ERR; percentage reduction in the number of eggs in the stool/urine after treatment, where the mean number of eggs from all people treated is calculated using either geometric or arithmetic means). We found that arithmetic and geometric means can be used interchangeably only if treatment efficacy is very high; arithmetic means are more sensitive to capture drops in efficacy expressed by ERR. A valid complement for drug efficacy monitoring is to study the distribution of individual responses in egg excretion that allows identifying in a single measure both those who had an adequate response to treatment and those who respond less well; e.g., the 5% of the patients with the lowest ERRs.

## Introduction

Schistosomiasis is a parasitic disease caused by blood flukes of the genus *Schistosoma*. The three main species infecting humans are *S*. *haematobium* (causing urogenital schistosomiasis), *S*. *japonicum*, and *S*. *mansoni* (the latter two responsible for intestinal schistosomiasis) [1]. The backbone of the global strategy for controlling the morbidity caused by schistosomiasis is the periodic administration of single-dose oral praziquantel (usually given at 40 mg/kg body weight). This strategy is termed “preventive chemotherapy”, whereby praziquantel is administered without prior diagnosis [2,3] to entire communities or target groups, most importantly school-aged children [4–6], depending on the level of endemicity [7,8].

How efficacy of antischistosomal drugs (and anthelmintic treatments in general) should be measured has been, and still is, a matter of debate in the research and disease control communities. One limitations of the current treatment outcome measure–i.e., parasite egg excretion–is that it is a proxy for drug effects on adult worms, which could also be confounded by various factors, including facultative temporary cessation of excretion by the adult worm [3]. Other, more direct but not yet widely used methods of worm vitality are the detection of specific antigens, like the circulating cathodic antigen (CCA) and the circulating anodic antigen (CAA) [9,10]. Clinical trials have traditionally used cure rates (CRs) as the main drug efficacy endpoint, and expressed results as the proportion of infected individuals who convert to a negative stool or urine sample post-treatment [11–13]. However, the World Health Organization (WHO) recently issued guidelines that recommend the egg reduction rate (ERR) as the primary outcome measure, especially when assessing programmatic treatment effectiveness [3]. This entails a quantitative diagnostic test based on the microscopic detection and enumeration of parasite eggs in small amounts of stool (usually 41.7 mg) and the estimation of the number of eggs per 1 g (EPG) of feces (*S*. *japonicum* or *S*. *mansoni*) or per 10 ml of urine (*S*. *haematobium*). The ERR measures the overall effect of treatment on the entire group of infected subjects treated (ignoring individual variability) and is expressed as the ratio between the mean of the pre- and post-treatment egg counts [3]. ERR is considered more suitable than CR to assess the impact of preventive chemotherapy on morbidity (which is commensurate to infection intensity) in the context of continuous risk of reinfection and in view of the low sensitivity of current diagnostic methods [12,14].

Which type of means (e.g., geometric mean (GM) or arithmetic mean (AM)) should be used to express treatment outcomes against helminthiases at the community level is an additional subject of debate [15,16]. Thus far, studies of treatment efficacy have predominantly reported results using GM egg counts [15] but recently, the use of AM egg counts has been advocated [17,18]. The issue is that egg counts are not normally distributed, even after logarithmic transformation, which would call for using GM [19]. However, GM hide extreme values (e.g., (i) a small proportion of individuals disproportionally contributing to total egg excretion, and (ii) individuals who do not respond to treatment), which are important when assessing the effects of interventions, and which are better captured by using AM.

A further complication when dealing with different studies is the diversity in methodologies, in particular: (i) which diagnostic method is used (e.g., single or multiple urine filtration for detection of *S*. *haematobium* eggs) [20] or the Kato-Katz technique for detection of *S*. *japonicum* or *S*. *mansoni* eggs in fecal samples, whose sensitivity depends upon the baseline infection intensity, the number of thick smears from a single sample, and the number of stool specimens examined [21–24]; and (ii) how many weeks post-treatment effects are measured, which also depends on *Schistosoma* species [25,26].

The objectives of this paper were to compare customary methods to assess the efficacy of praziquantel for treating schistosomiasis and to explain differences; to identify possible alternative approaches to express treatment effects on egg excretion; and to verify whether the size of treatment effects for intestinal schistosomiasis change when measured with a single or quadruplicate Kato-Katz thick smears. The overall aim of these analyses was to derive suggestions for standardizing outcome measures in future drug efficacy studies. For this purpose, we combined and analyzed available data from various studies where praziquantel was used to treat infections with different *Schistosoma* species.

## Methods

### Ethics Statement

All studies selected for the current secondary analyses had been approved by the relevant institutional review boards and ethics committees, and were conducted according to international ethics standards (for details, see individual publications [25–36]). Data received from the individual studies were completely anonymized.

### Datasets Analyzed

We built a common database from 24 studies including 4,740 individuals assigned to three different treatment groups who had received either 40 mg/kg (18 studies, 3,713 individuals), 60 mg/kg (five studies, 690 individuals), or 80 mg/kg (one study, 337 individuals) praziquantel against *S*. *haematobium* [25,27–29,34,36], *S*. *japonicum* [31,32], and *S*. *mansoni* [21,23,26–28,30,31,33,35].

The main study characteristics, including *Schistosoma* species, praziquantel dose, age of participants, time-point of treatment follow-up, and diagnostic approach, are summarized in Table 1. Studies enrolled a total of 4,740 individuals, of whom 2,633 (55.5%) were infected with *S*. *haematobium*, 1,804 (38.1%) with *S*. *mansoni*, and the remaining 303 (6.4%) with *S*. *japonicum*. Studies generally enrolled children and adolescents except one study in the People’s Republic of China [32] and another one in Côte d’Ivoire [33]. The praziquantel dose was 40 mg/kg in 17 studies, 60 mg/kg in five studies, and 80 mg/kg in one study. Follow-up was within 3 weeks in 13 studies, four weeks in two studies, within 2 months in six studies, and longer in the remaining three studies.

**Table 1:** Study and individuals’ main characteristics.

Study, year [reference]	Species^1^	Praziquantel dose (mg/kg)	Age^2^	Follow-up (days)	Diagnostic approach at pre- and post-treatment
**Côte d’Ivoire, 2011 [28]**	Sh	40	3.7 ± 1.1	21	Single filtration per urine sample
**Niger, 2007 [27]**	Sh	40	9.1 ± 2.2	42	Three consecutive urine samples over three days, filtered once
**Niger, 2009 [27]**	Sh	40	10.1 ± 2.3	21	Three consecutive urine samples over three days, filtered once
**Côte d’Ivoire, 2000 [34]**	Sh	80	9.5 ± 2.6	52	Single filtration per urine sample
**Mali, 2006 [29]**	Sh	40	10.4 ± 2.3	182.5	Single filtration per urine sample
**Mali, 2009 [29]**	Sh	40	3.6 ± 1.2	365	Single filtration per urine sample
**Côte d’Ivoire, 2010 [25]**	Sh	40	11.0 ± 2.2	21	Single filtration per urine
**Kenya, 1990 [36]**	Sh	40	11.2 ± 3.3	45	Duplicate filtration per urine sample × 2 samples
**Philippines, 2007 [31]**	Sj	40	12.5 ± 2.0	21	Duplicate KK per stool sample × 2 samples
**Philippines, 2007 [31]**	Sj	60	12.4 ± 2.0	21	Duplicate KK per stool sample × 2 samples
**People’s Republic of China, 2007 [32]**	Sj	40	46.1 ± 14.5	90	Triplicate KK per stool sample × 3 samples
**Côte d’Ivoire, 2011 [28]**	Sm	40	3.8 ± 1.2	21	Duplicate KK per stool sample × 2 samples
**Uganda, 2012 [30]**	Sm	40	4.1 ± 1.7	42	Duplicate KK per stool sample × 2 samples
**Brazil, 2007 [31]**	Sm	40	15.2 ± 2.8	21	Duplicate KK per stool sample × 2 samples
**Brazil, 2007 [31]**	Sm	60	14.9 ± 2.5	21	Duplicate KK per stool sample × 2 samples
**Mauritania, 2007 [31]**	Sm	40	12.6 ± 2.0	21	Duplicate KK per stool sample × 2 samples
**Mauritania, 2007 [31]**	Sm	60	12.6 ± 2.1	21	Duplicate KK per stool sample × 2 samples
**Tanzania, 2007 [31]**	Sm	40	12.3 ± 1.8	21	Duplicate KK per stool sample × 2 samples
**Tanzania, 2007 [31]**	Sm	60	12.7 ± 2.1	21	Duplicate KK per stool sample × 2 samples
**Côte d’Ivoire, 2004 [33]**	Sm	40	21 ± 49.4	42	Single KK per stool sample × 3 samples
**Côte d’Ivoire, 2007 [26]**	Sm	40	8.9 (5–13)*	20	Duplicate KK per stool sample × 2 samples
**Côte d’Ivoire, 1997 [35]**	Sm	40	9.3 (6–14)*	28	Single KK per stool sample × 4 samples
**Côte d’Ivoire, 1998 [35]**	Sm	60	10 (7–14)**	28	Five consecutive stool samples (1 KK per sample) at pre- treatment and four consecutive stool samples (3 KK per samples) at post- treatment

^1^Species: Sh = *Schistosoma haematobium*, Sj = *Schistosoma japonicum*, Sm = *Schistosoma mansoni*.

^2^Age is expressed as the mean ± standard deviation, except for *mean (range) and ** median (range).

For the detection of *S*. *haematobium* infection, two diagnostic approaches were employed: (i) a single urine filtration slide in five studies (one of them was carried out on the same sample but at two different time points) [25,26,28,29,34]; and (ii) duplicate urine filtration slides in one study [36]. For the diagnosis of *S*. *japonicum*, duplicate Kato-Katz thick smears from each of two stool specimens were subjected to microscopic examination in all studies. For the detection of *S*. *mansoni*, the most common diagnostic approach was duplicate Kato-Katz thick smears from each of two stool specimens. In one study, a single Kato-Katz thick smear was performed on five samples at baseline and triplicate Kato-Katz thick smears for four samples at follow-up [35,37].

### Efficacy Outcomes and Calculations

Treatment response was assessed both at the overall group and the individual level. The AM and GM EPG values were calculated at pre- and post-treatment for S. mansoni and S. japonicum by multiplying the individual fecal egg counts (FECs) obtained by a single Kato-Katz thick smear (41.7 mg) by a factor of 24. For S. haematobium, egg counts are presented as eggs per 10 ml of urine. Drug efficacy was expressed as ERR and CR.

ERR (arithmetic (ERR_AM_) or geometric (ERR_GM_)) was calculated as the ratio of the difference between the (arithmetic or geometric) means of the pre- and post-treatment EPG or eggs per 10 ml urine to the pre-treatment (arithmetic or geometric) mean EPG or eggs per 10 ml urine: ERR = [(mean egg count_pre-treatment_ − mean egg count_post-treatment_) / mean egg count_pre-treatment_] x 100

GM egg counts were calculated as follows: $\text{exp}\left[\frac{\sum_{i=1}^{i=n_{jk}}\text{ln}\left(x_{ijk}+c\right)}{n_{jk}}\right]-c$, where *x*_*ijk*_ is the observed egg count for individual host *i*, *Schistosoma* species *j*, and study *k*; *n*_*jk*_ is the number of hosts who provided a (pre- and post-treatment) sample for determination of infection intensity for each *Schistosoma* species and study, and *c* is a constant added to each count to allow inclusion of zero counts (negative test) [38]. Confidence intervals (CIs) for the ERR (calculated with AM and GM) were determined by using a bootstrap resampling method (with replacement) over 1,000 replicates and expressed as a univariate calculation of the 2.5^th^ and 97.5^th^ percentiles.

Individual ERR was calculated as the ratio of the difference between the pre- and post-treatment EPG or eggs per 10 ml urine to the pre-treatment EPG or eggs per 10 ml urine multiplied by 100.

CRs and 95% binomial CIs were the percentage of infected individuals negative for *Schistosoma* (in their urine or stool) at post-treatment follow-up. The distribution of individual responses in egg excretion was categorized as (i) negative (corresponding to CR), (ii) reduction, (iii) no change or increase, and further expressed in centiles to quantitate the fraction of poor responders.

### Comparing Methods of Assessing Drug Efficacy

We compared (i) the ERR_AM_ versus ERR_GM_ and (ii) the CR versus ERR. The results were presented graphically in modified L’Abbé plots with 95% CIs for both comparisons, and additionally in Bland and Altman plots for ERR using AM and GM. The coefficient of determination (R^2^) was also calculated.

A linear mixed model of the difference of the ERRs calculated as GM and AM (ΔERR_g,a_) was developed to estimate which parameter could better predict the difference between the two ERR calculations, with the average of the two mean ERRs (GM and AM) set as covariate. Such a model was extrapolated from the Bland and Altman regression by further including predictive factors. In order to evaluate the effect of different factors on ΔERR_g,a_, the ERR was calculated on the different strata defined by the combination of the categories of the following parameters: age, sex, treatment dose, and *Schistosoma* species. The same age categories were defined across all studies. The linear mixed model was estimated including these parameters as independent factors. 95%CIs of the difference between the two ERR calculations were calculated by using Tango’s score confidence interval which was shown in the literature to outperform other calculation methods in the case of correlated proportions [39,40].

Modeling was carried out through a shrinkage method of variable selection. Variables were first selected using the ElasticNet procedure, which is mixing a least absolute shrinkage and selection operator (LASSO) procedure and ridge regression [41]. Subsequently, a variance-covariance matrix structure was selected among unstructured, variance components, autoregressive, compound symmetry and Toeplitz structures, which minimized the Aikake information criterion corrected (AICC) for finite sample size. Post-hoc tests on each parameter were carried out with a Tukey adjustment. Pairwise differences in least square means (LSM) were calculated for fixed values of the average of the AM and GM ERRs (for the range 70–99%), thereby evaluating the influence of exogenous parameters on the bias between the two ERRs. This bias was evaluated in the Bland and Altman method by regressing the difference of the two methods by their mean.

All tests were two-tailed; a p-value of 5% was deemed significant. Only studies with treatment follow-up examination done within 90 days were included in the models (to minimize the effect of reinfection after praziquantel administration). Calculations and analyses were performed by using SAS system version 9.3 (SAS Institute, Cary, NC, United States of America).

### Comparing Efficacy Assessments Based on Single versus Quadruplicate Kato-Katz Thick Smears for *S. mansoni*

For *S. mansoni*, we compared single (using the first thick smear on the first stool specimen) *versus* quadruplicate (using four thick smears of the same stool specimen) Kato-Katz thick smears for expressing CR and ERR (with AM and GM). In this sub-analysis, we only included individuals infected with S. mansoni whose first slide on the first fecal sample was positive.

Within this population, we calculated and compared the overall AM and GM pre- and post-treatment FECs and the respective ERRs and CRs based on single and quadruplicate Kato-Katz thick smears with 95% CIs calculated by boot-strapping. The results of the individual studies were presented graphically in Bland and Altman plots and in modified L’Abbé plots with 95% CIs, and the coefficient of determination (R^2^) was calculated.

## Results

### Study Characteristics

**Fig 1:**
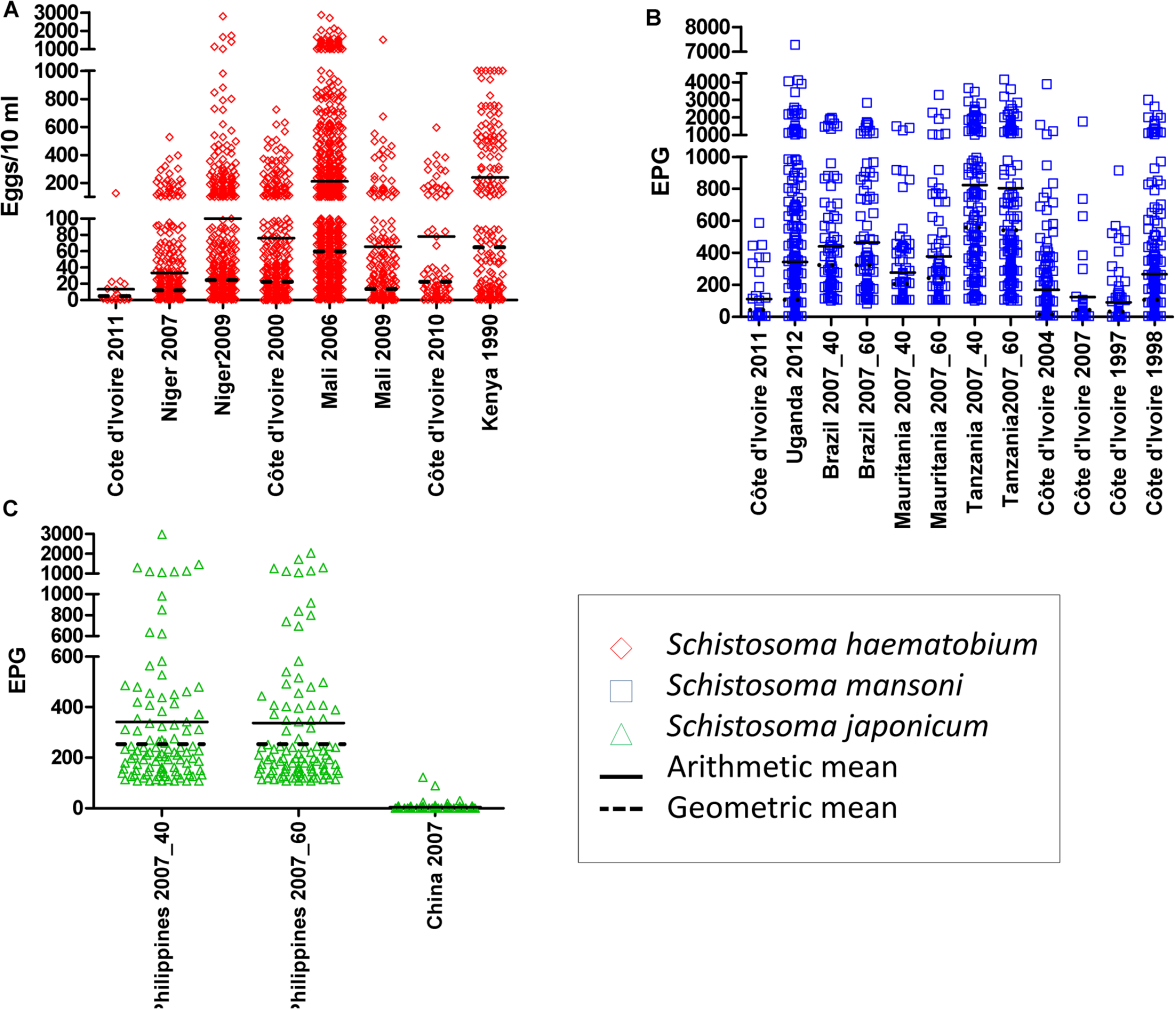
Distribution of raw egg count at baseline by *Schistosoma* species and study. A. *Schistosoma haematobium*. B. *Schistosoma mansoni*. C. *Schistosoma japonicum*.

The distributions of the raw egg counts at baseline by S*chistosoma* species for each study (including AMs and GMs) are presented in Fig 1. Efficacy outcomes were analyzed on a total of 4,375 individuals with pre- and post-treatment egg counts. Among them, 2,365 (54.1%) were infected with *S*. *haematobium*, 1,708 (39.0%) with *S*. *mansoni*, and the remaining 300 (6.9%) with *S*. *japonicum*. Details of baseline FECs and drug efficacy outcomes, including group-based means and individual responses by study and *Schistosoma* species, are presented in Table 2.

**Table 2:** Summary of study outcomes, group-based mean responses, and individual responses by study and species.

			Baseline egg counts		Group mean responses						Individual responses							** **
Study	Dose (mg/kg)	N	aMD0	gMD0	ERR_AM_	Lower limit 95%CI	Upper limit 95%CI	ERR_GM_	Lower limit 95%CI	Upper limit 95%CI	CR	Lower limit 95%CI	Upper limit 95%CI	ERR = 0%	iERR 5^th^ centile	iERR 10^th^ centile	iERR 25^th^ centile	ERR <90%
Côte d'Ivoire, 2011	40	18	13.44	4.34	99.38	99.00	100.00	98.80	96.81	100.00	94.4%	83.9%	100%	0.00%	0.0%	98.8%	98.8%	0.00
Niger, 2007	40	377	33.09	11.73	96.50	95.09	97.60	96.33	95.58	97.00	69.2%	64.6%	73.9%	0.53%	77.8%	88.1%	97.9%	11.14
Niger, 2009	40	401	95.99	25.52	95.73	92.23	98.14	96.72	95.90	97.30	51.4%	46.5%	56.3%	3.24%	42.5%	84.6%	96.4%	13.72
Côte d'Ivoire, 2000	80	331	76.19	24.75	99.82	99.68	99.93	99.76	99.61	99.87	91.5%	88.5%	94.5%	0.30%	99.7%	100.0%	100.0%	0.91
Mali, 2006	40	829	218.86	67.13	92.48	90.29	94.30	97.41	96.90	97.82	59.1%	55.8%	62.5%	5.43%	0.0%	50.0%	95.1%	19.90
Mali, 2009	40	162	67.01	16.57	18.94	-19.93	45.07	58.08	39.31	71.80	70.4%	63.3%	77.4%	38.89%	0.0%	0.0%	0.0%	56.79
Côte d'Ivoire, 2010	40	87	85.53	27.29	97.83	95.88	99.31	98.58	97.58	99.32	80.5%	72.1%	88.8%	1.15%	82.4%	95.0%	99.5%	4.60
Kenya, 1990	40	162	238.94	69.42	55.20	41.15	67.96	90.67	85.95	94.10	65.4%	58.1%	72.8%	14.20%	0.0%	0.0%	31.9%	42.59
***S*. *haematobium***		**2367**	**133.02**	**32.65**	**86.64**	**84.03**	**89.07**	**96.16**	**95.72**	**96.54**	**61.3%**	**59.3%**	**63.3%**	**6.25%**	**0.0%**	**49.3%**	**96.5%**	**18.17**
Philippines, 2007	40	101	339.57	251.54	99.58	99.20	99.87	99.91	99.83	99.97	93.1%	88.1%	98.0%	0.00%	97.4%	98.8%	98.8%	1.98
Philippines, 2007	60	99	337.15	248.70	99.96	99.87	100.00	99.99	99.96	100.00	99.0%	97.0%	100%	0.00%	96.8%	96.8%	96.8%	0.00
China, 2007	40	100	4.48	1.42	83.55	48.80	96.43	86.95	78.28	93.62	78.0%	69.9%	86.1%	5.00%	0.0%	25.6%	99.0%	19.00
***S*. *japonicum***		**300**	**227.08**	**52.45**	**99.66**	**99.42**	**99.87**	**99.73**	**99.55**	**99.86**	**90.0%**	**86.6%**	**93.4%**	**1.67%**	**75.0%**	**99.0%**	**99.0%**	**7.00**
Côte d'Ivoire, 2011	40	35	111.34	36.41	99.38	98.45	100.00	99.44	98.52	100.00	91.4%	82.2%	100%	0.00%	50.0%	96.8%	96.8%	5.71
Uganda, 2012	40	305	332.73	104.31	82.17	72.69	89.03	96.28	94.95	97.22	56.4%	50.8%	62.0%	10.82%	0.0%	0.0%	81.1%	30.16
Niger, 2007	40	183	4230.60	2781.20	81.46	75.93	86.77	99.38	98.93	99.63	59.6%	52.5%	66.7%	7.10%	0.0%	18.2%	63.2%	36.61
Brazil, 2007	40	96	441.56	308.73	100.00	100.00	100.00	100.00	100.00	100.00	100%	100%	100%	0.00%	0.0%	0.0%	0.0%	0.00
Brazil, 2007	60	94	476.55	326.29	99.99	99.95	100.00	99.99	99.98	100.00	98.9%	96.9%	100%	0.00%	97.4%	97.4%	97.4%	0.00
Mauritania, 2007	40	92	278.26	210.63	98.78	96.82	99.87	99.88	99.76	99.96	92.4%	87.0%	97.8%	0.00%	96.0%	97.2%	97.2%	1.09
Mauritania, 2007	60	93	378.41	238.95	94.02	85.08	99.89	99.87	99.72	99.96	92.5%	87.1%	97.8%	0.00%	97.2%	99.4%	99.4%	3.23
Tanzania, 2007	40	119	813.53	565.01	95.88	91.73	99.01	99.87	99.77	99.94	86.6%	80.4%	92.7%	1.68%	73.2%	97.8%	99.7%	6.72
Tanzania, 2007	60	125	838.82	558.34	98.74	96.99	99.68	99.91	99.83	99.95	88.0%	82.3%	93.7%	0.00%	92.5%	98.1%	99.8%	2.40
Côte d'Ivoire, 2004	40	177	168.86	56.62	83.70	72.85	91.54	95.89	94.18	97.08	62.1%	55.0%	69.3%	7.34%	0.0%	31.3%	90.9%	23.73
Côte d'Ivoire, 2007	40	49	111.58	31.74	87.06	49.89	99.17	98.12	95.29	99.54	87.8%	78.6%	96.9%	8.16%	0.0%	0.0%	84.6%	12.24
Côte d'Ivoire, 1997	40	85	91.79	27.20	96.06	93.17	98.33	97.20	95.48	98.35	70.6%	60.9%	80.3%	3.53%	40.5%	81.1%	97.9%	12.94
Côte d'Ivoire, 1998	60	255	255.60	95.57	93.62	88.72	96.87	98.58	98.10	98.99	72.9%	67.5%	78.4%	2.35%	43.8%	82.8%	98.3%	12.94
***S*. *mansoni***		**1708**	**783.11**	**190.87**	**86.59**	**83.31**	**89.65**	**99.12**	**98.98**	**99.24**	**75.2%**	**73.2%**	**77.3%**	**4.33%**	**18.2%**	**65.3%**	**99.8%**	**15.75**

N is the number assessed (egg counts at pre- and post-treatment) out of the total number enrolled. Group means by species are calculated by pooling all studies on that species. 95% confidence interval (CI) are calculated by boot-strapping. Egg reduction rate (ERR) = 0 is the proportion of subjects who had no reduction in egg counts; the proportions in the 5^th^, 10^th^, and 25^th^ centile columns represent the individual ERR achieved by that centile.

### Comparing ERRs Obtained with AM versus GM

ERR_AM_ ranged from 17.0% to 99.8% and ERR_GM_ from 50.7% to 99.8% for *S. haematobium*; 81.5–100% (ERR_AM_) and 95.9–100% (ERR_GM_) for *S. mansoni*; 83.5–99.9% (ERR_AM_) and 86.9–99.9% (ERR_GM_) for *S. japonicum*. The 95% CIs estimated by boot-strapping tended to be wider with AM compared to GM. Among the 24 studies included in our analyses, six had an ERR <90% by AM (two if restricted to studies with 3 weeks’ follow-up), but only two by GM.

**Fig 2:**
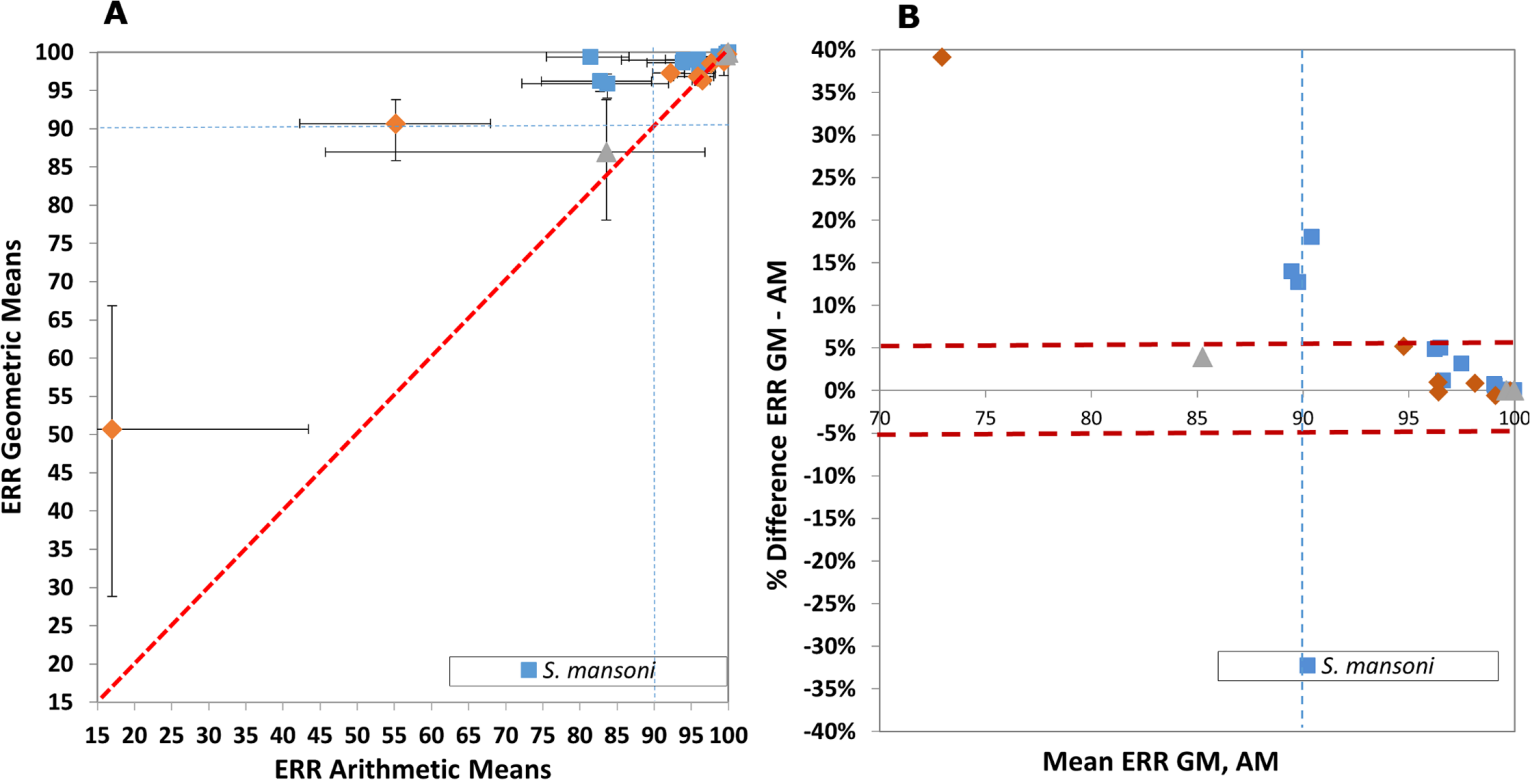
Comparison of egg reduction rate calculated as arithmetic mean (ERR_AM_) *versus* geometric mean (ERR_GM_), both with estimated 95% confidence intervals (CIs). A. L’Abbé plot, B. Bland & Altman plot. For each point, the 95% CIs are shown in the L’Abbé plot, where the red dashed diagonal line of no difference between the ERR calculated with both metrics. The blue dashed line represents the threshold for acceptable efficacy defined by WHO [3].

The modified L’Abbé plot (Fig 2A) indicates that ERRs tend to be higher when calculated using GM. The R^2^ of the linear regression showed a strong linear correlation for *S*. *japonicum* (R^2^ = 1.00, three studies) and *S*. *haematobium* (R^2^ = 0.88, eight studies), but a weaker linear correlation for *S*. *mansoni* (R^2^ = 0.46, 13 studies).

### Modeling Group-Based ERRs

The mixed linear model found a significant relationship between the difference between ERR_AM_ and ERR_GM_ and their mean value. However, introducing the variables identified as predictors by the ElasticNet procedure, coupled with model averaging based on 1000 replicates (sampling with replacement)–mean baseline epg, year of study and species–rendered this relation non-significant (meaning that none of these covariates could explain the differences between ERR_AM_ and ERR_GM_.)

LSM pairwise comparisons and ERR (individually for AM and GM) models showed a significantly better consistency between ERRs calculated with AM and GM for *S*. *haematobium* than for *S*. *mansoni*. Study participants’ age was found to have an effect only for ERR_GM_ (higher for school-aged children and adults than for preschool-aged children; S1 Table).

### Distribution of Individual Treatment Responses in Egg Excretion

**Fig 3:**
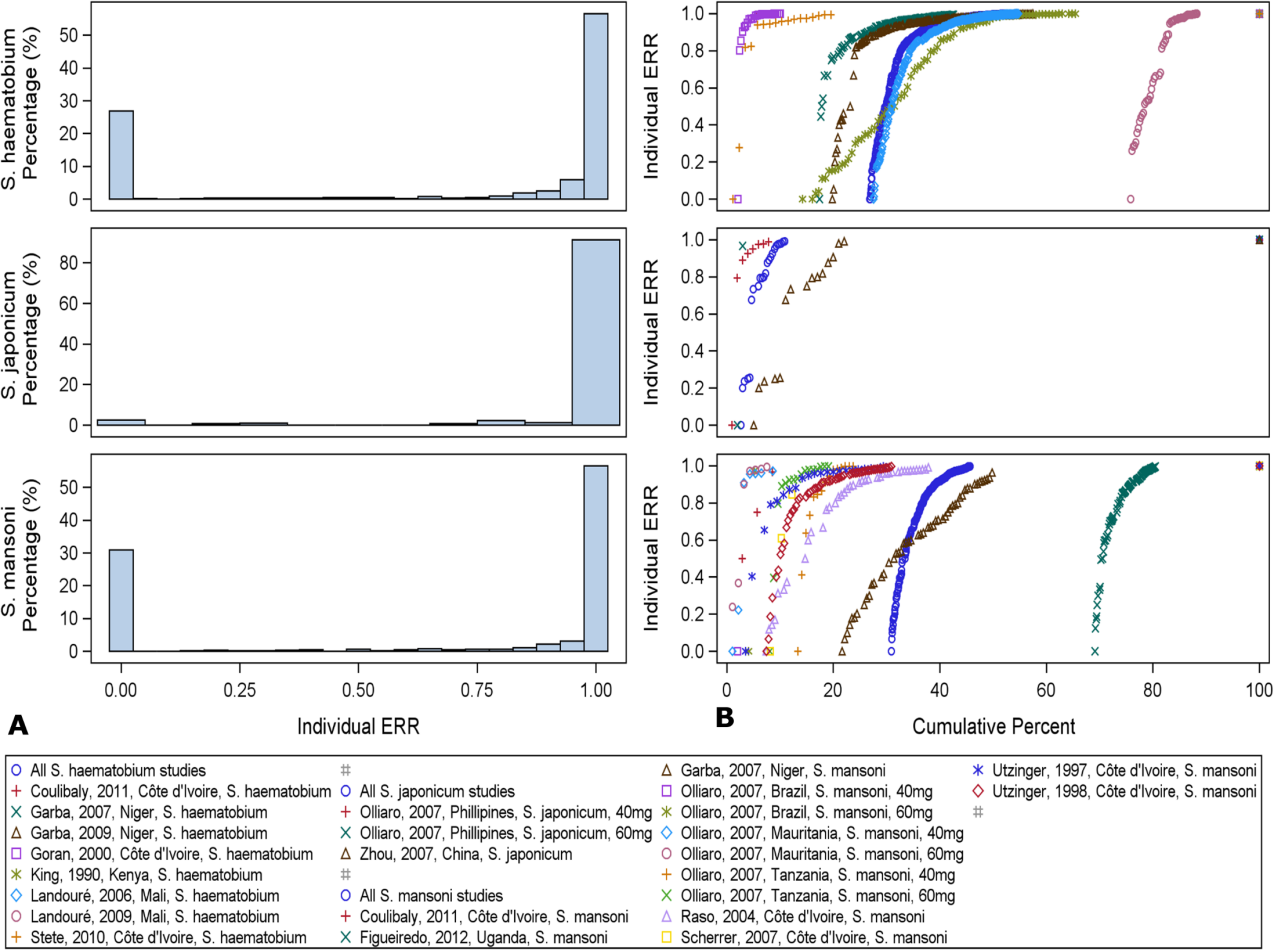
Distribution of individual ERR. A. Bar diagrams, B. Centile distribution.

Group means and individual responses are presented in Table 2. Individual responses are also displayed graphically in Fig 3 (panel A as bar diagrams; panel B as centile distributions). Overall, 6.3% (ranging in individual studies from 0 to 39%), 1.7% (0–5%); and 4.3% (0–11%) of the subjects treated for *S*. *haematobium*, *S*. *japonicum*, and *S*. *mansoni* infection, respectively, had no reduction in their egg counts (ERR = 0). The 5^th^, 10^th^, and 25^th^ centiles of the subjects treated for *S*. *haematobium* had individual ERRs of 0% (ranging in individual studies from 0 to 99.7%), 49.3% (0–100%), and 96.5% (0–100%); the corresponding values for *S*. *japonicum* were 75.0% (0–97.4%), 99.0% (25.6–98.8%), and 99.0% (96.8–99%), and for *S*. *mansoni* 18.2% (0–97.4%), 65.3% (0–99.4%), and 99.8% (0–99.8%).

**Fig 4:**
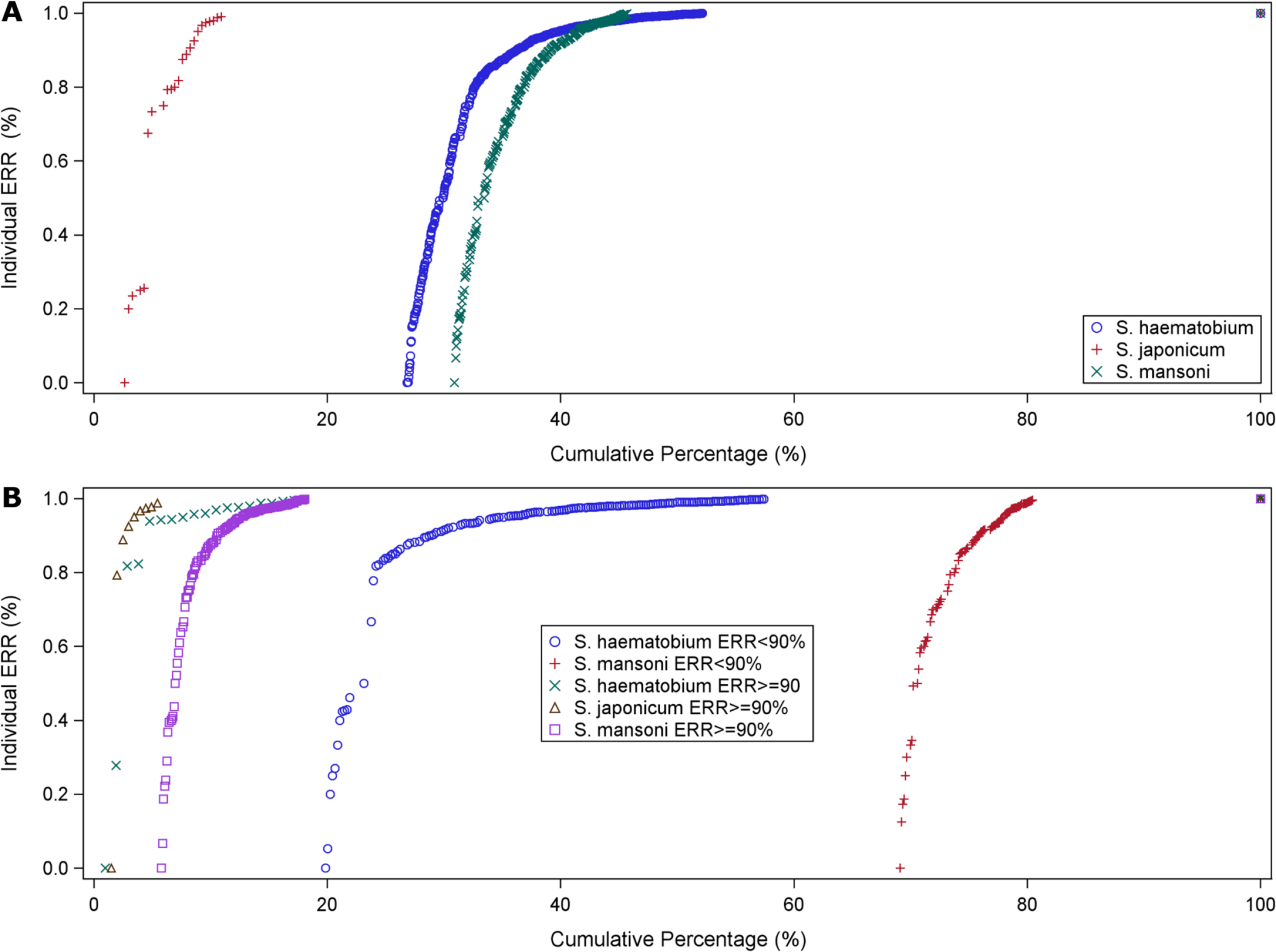
Centile distribution of individual ERRs by species for all studies (A) and for studies with assessment within 28 days divided by ERR_AM_ ≥90% and <90% (B).

The centile distribution of iERRs in studies with ERR_AM_ <90% was shifted to the right and clearly distinct from those with ERR_AM_ ≥90% (Fig 4).

When considering only studies assessing outcomes within a maximum of 28 days (13 studies evaluated drug efficacy at 21 days, two at 28 days), only two (both treating *S*. *mansoni* with 40 mg/kg praziquantel with 21-day follow-up [27,30]) had an ERR_AM_ <90%; in terms of individual responses, the 30^th^ and 36^th^ centile, respectively had ERRs <90%, and 10.8% and 7.1% of patients, respectively had no change in their egg counts (ERR = 0). Three additional studies, all with ERR AM >90%, had individual ERRs <90% in the 13^th^ centile: one study on *S*. *haematobium* treated with 40 mg/kg and 21-day follow-up [27], and two studies on *S*. *mansoni* treated with 40 or 60 mg/kg with 28-day follow-up [35,37]. In these studies, 3.2%, 3.5% and 2.4%, respectively of individuals had no decrease in egg counts. Across these studies, ERR_AM_ and iERR were highly correlated (R^2^ 0.95).

### Comparing CR versus ERR

**Fig 5:**
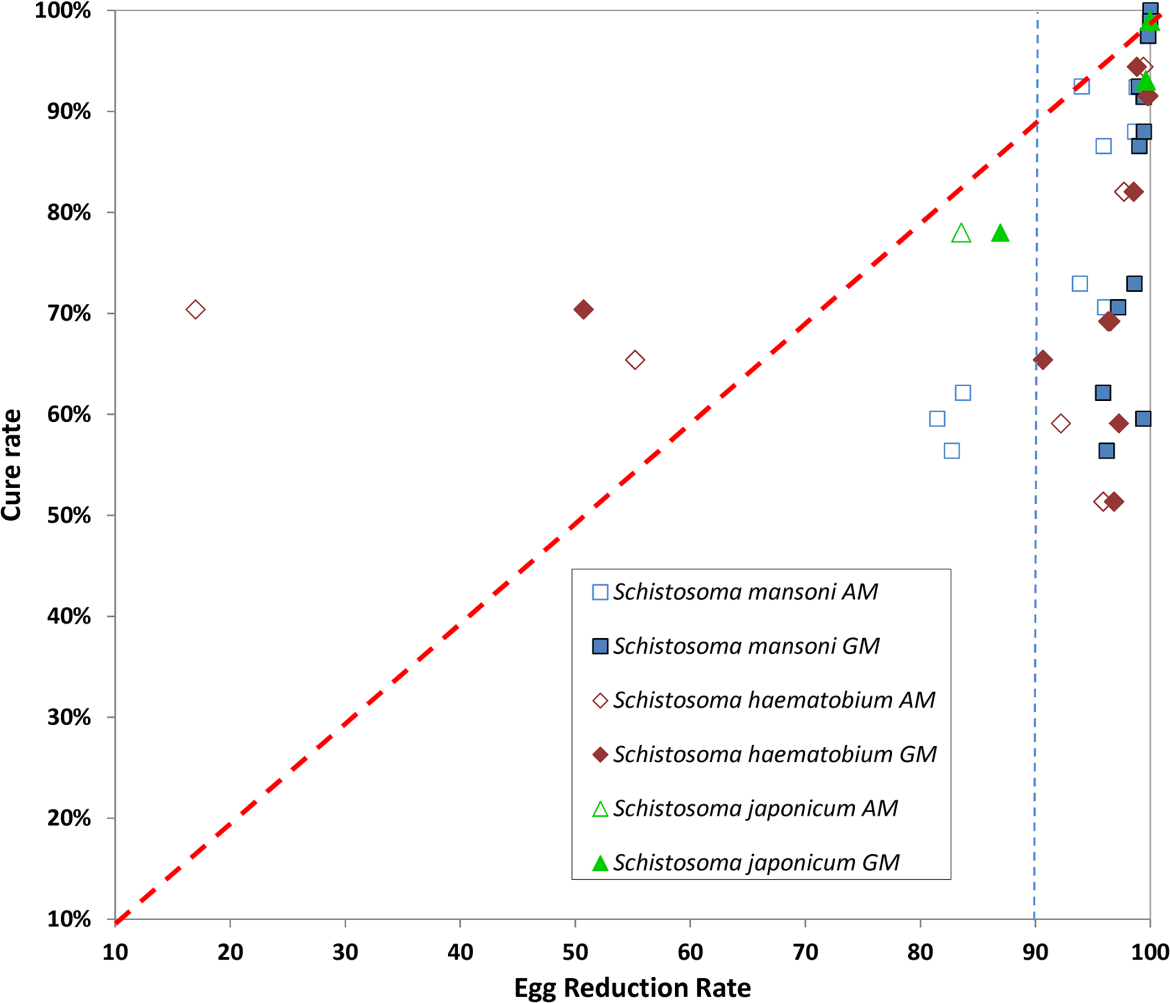
Comparison between egg reduction rate (ERR) and cure rate (CR). The red dashed diagonal line of no difference between the ERR calculated with both metrics.

For all three *Schistosoma* species, the CR was systematically lower than ERR, regardless of whether AM or GM was employed, except in a single study [29]. ERR >90% corresponded to CRs ranging from 51.4% to 99%. Only when ERRs were very high (range: 97.7–100%) there was a good agreement between both indicators. The CR ranged from 82.1% to 100% (Fig 5).

### Multiple versus Single Kato-Katz Thick Smear Examination for *S. mansoni*

**Fig 6:**
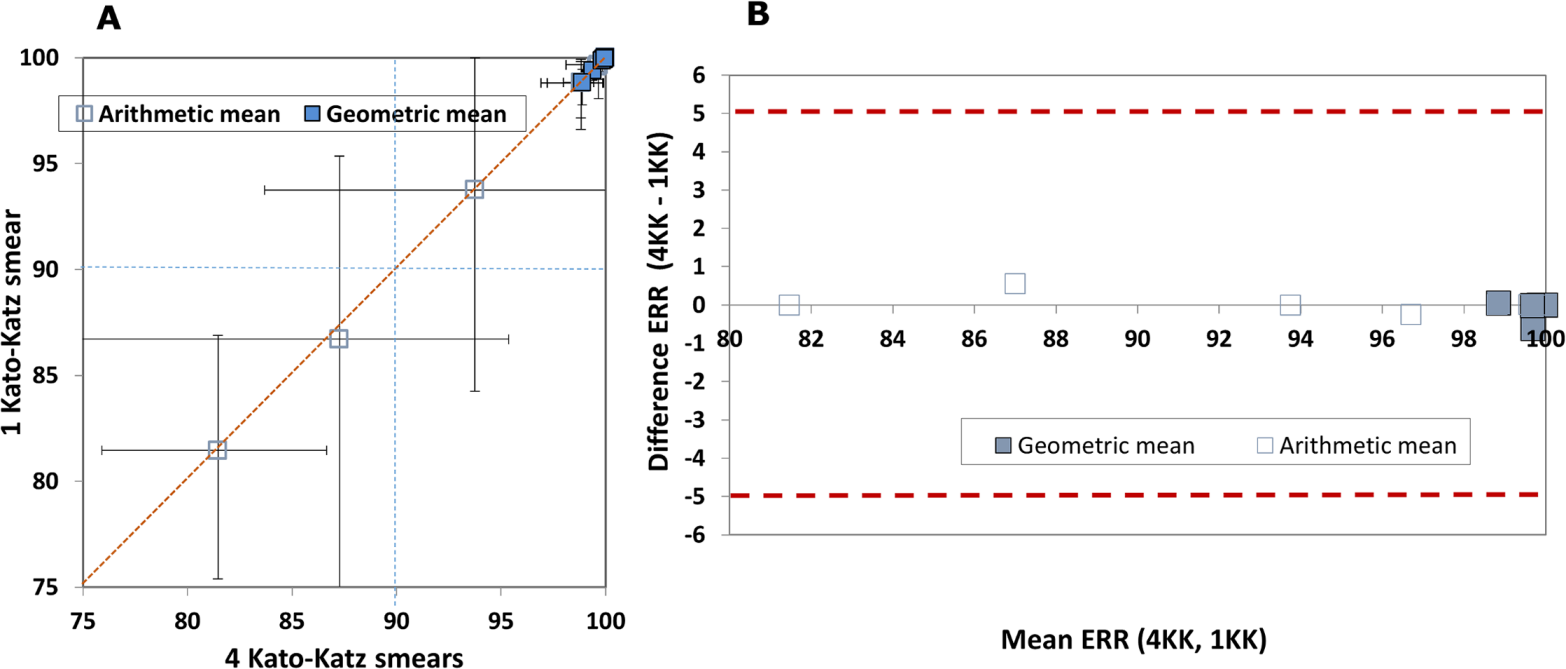
Comparison of egg reduction rate (ERR) calculated from a single *versus* quadruplicate Kato-Katz thick smears, both with estimated 95% confidence intervals (CIs). A. L’Abbé plot. For each point, the 95% CIs are shown. The red dashed diagonal line of no difference between the ERR calculated with both metrics. The blue dashed line represents the threshold for acceptable efficacy defined by the WHO [3]. B. Bland-Altman plot. The red dashed lines show the interval that determines the agreement of the two metrics, set at 5%. The blue dashed line represents the threshold for acceptable efficacy defined by the WHO [3].

Among the 1,435 individuals enrolled in the studies who were found positive for *S. mansoni* eggs in their stool based on quadruplicate Kato-Katz thick smears, 1,167 (81.3%) were diagnosed positive on the first Kato-Katz thick smear. In this subset, we found that the results in the individual studies were highly correlated (Fig 6A–6B; R^2^ = 0.95 for AM and R^2^ = 0.86 for GM). The same number of studies (n = 2) had AM ERR of less than 90% with either approach.

On aggregate, the FECs at baseline were lower with quadruplicate than with a single Kato-Katz thick smear when using either AM (1,046 EPG *versus* 2,617 EPG) or GM (342 EPG *versus* 575 EPG); the ERR estimates were comparable only by GM (99.5% *versus* 99.8%) whereas by AM, the estimate was lower with quadruplicate than single examinations (86.9% *versus* 94.9%) (Table 3). In order to verify if using a single Kato-Katz thick smear selected for a different sample, we compared the baseline FECs and ERRs assessed with quadruplicate Kato-Katz thick smears on the overall sample of these studies (n = 1,435) to that of this subgroup with the first Kato-Katz thick smear positive (n = 1,167): while the overall average baseline FECs was approximately threefold lower for the latter, there was no difference in ERR between the two groups for either AM and GM.

**Table 3:** Data outcomes at baseline in individual positives on single or quadruplicate Kato-Katz thick smears.

Study	Species	Dose (mg/kg)	% subjects with ERR = 0%	% subjects with iERR <90%	iERR 5^th^ centile	iERR 10^th^ centile	iRR 25^th^ centile	ERR_AM_^(^ ^1^ ^)^	Lower limit 95%CI^(^ ^2^ ^)^	Upper limit 95%CI^(^ ^2^ ^)^
Côte d'Ivoire, 2001	*S*. *haematobium*	40	0.0%	0.0%	0.0%	98.8%	98.8%	99.4%	99.0%	100.0%
Niger, 2009	*S*. *haematobium*	40	3.2%	13.7%	42.5%	84.6%	96.4%	95.7%	92.2%	98.1%
Côte d'Ivoire, 2010	*S*. *haematobium*	40	1.1%	4.6%	82.4%	95.0%	99.5%	97.8%	95.9%	99.3%
Philippines, 2007	*S*. *japonicum*	40	0.0%	2.0%	97.4%	98.8%	98.8%	99.6%	99.2%	99.9%
Philippines, 2007	*S*. *japonicum*	60	0.0%	0.0%	96.8%	96.8%	96.8%	100.0%	99.9%	100.0%
Uganda, 2012	*S*. *mansoni*	40	10.8%	30.2%	0.0%	0.0%	81.1%	82.2%	72.7%	89.0%
Niger, 2007	*S*. *mansoni*	40	7.1%	36.6%	0.0%	18.2%	63.2%	81.5%	75.9%	86.8%
Brazil, 2007	*S*. *mansoni*	40	0.0%	0.0%	0.0%	0.0%	0.0%	100.0%	100.0%	100.0%
Brazil, 2007	*S*. *mansoni*	60	0.0%	0.0%	97.4%	97.4%	97.4%	100.0%	100.0%	100.0%
Mauritania, 2007	*S*. *mansoni*	40	0.0%	1.1%	96.0%	97.2%	97.2%	98.8%	96.8%	99.9%
Mauritania, 2007	*S*. *mansoni*	60	0.0%	3.2%	97.2%	99.4%	99.4%	94.0%	85.1%	99.9%
Tanzania, 2007	*S*. *mansoni*	40	1.7%	6.7%	73.2%	97.8%	99.7%	95.9%	91.7%	99.0%
Tanzania, 2007	*S*. *mansoni*	60	0.0%	2.4%	92.5%	98.1%	99.8%	98.7%	97.0%	99.7%
Côte d'Ivoire, 1997*	*S*. *mansoni*	40	3.5%	12.9%	40.5%	81.1%	97.9%	96.1%	93.2%	98.3%
Côte d'Ivoire, 1998*	*S*. *mansoni*	60	2.4%	12.9%	43.8%	82.8%	98.3%	93.6%	88.7%	96.9%

• follow-up 28 days

^1^Egg reduction rate (ERR).

^2^95% confidence interval (CI) calculated using a bootstrap resampling method.

**Fig 7:**
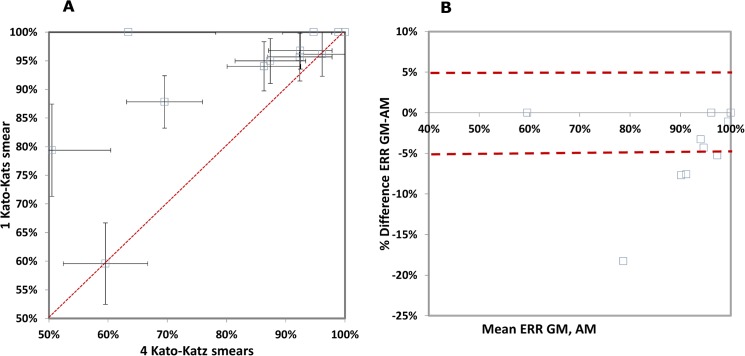
Comparison of cure rate (CR) calculated from a single *versus* quadruplicate Kato-Katz thick smears, both with estimated 95% confidence intervals (CI). A. L’Abbé plot. For each point, the 95% CIs are shown. The red dashed diagonal line of no difference between the ERR calculated with both metrics. B. Bland-Altman plot. The red dashed lines show the interval that determines the agreement of the two metrics, set at 5%.

In contrast to ERRs, CRs derived from single *versus* quadruplicate Kato-Katz thick smears were poorly correlated (R^2^ = 0.50) (Fig 7). With few exceptions, the CR assessed from single Kato-Katz thick smear (CR = 92.0%) was consistently higher than that from quadruplicate Kato-Katz thick smears (CR = 82.6%) in the individual studies and the difference between the two approaches increased with decreasing efficacy (Fig 7A and 7B). The overall CR was 88.0% applying a single and 79.0% using quadruplicate Kato-Katz thick smears (Table 4).

**Table 4:** Drug efficacy outcomes at baseline in individuals detected positive for *S*. *mansoni* by a single or quadruplicate Kato-Katz thick smear examinations at baseline.

Sample screened	Kato-Katz examination	n	^1^AMD0	^2^GMD0	^3^ERR AM (± 95% ^4^CI)	ERR GM (± 95% ^4^CI)	^5^CR (± 95% CI)
**Overall sample positive on 4** ^**6**^ **KK examinations**	4 KK	1,435	878.7	223.7	86.9 (83.6–90.0)	99.4 (99.3–99.5)	79.6 (77.4–81.7)
**Subgroup positive on 4 and 1 KK examinations**	4 KK	1,167	1046.3	342.1	86.9 (83.3–90.6)	99.6 (99.4–99.6)	79.0 (76.7–81.3)
	1 KK	1,167	2617.5	574.8	94.92 (93.18–96.39)	99.83 (99.78–99.86)	88.1 (86.2–89.9)

^1^Arithmetic mean (AM) at baseline

^2^Geometric mean (GM) at baseline

^3^Egg reduction rate (ERR)

^4^95% confidence interval (CI) calculated using a bootstrap resampling method

^5^Cure rate (CR).

^6^Kato-Katz.

## Discussion

Preventive chemotherapy with praziquantel is the current backbone of the global strategy to control the morbidity caused by schistosomiasis in high-endemicity areas [2,4,6,8]. Monitoring of praziquantel efficacy should accompany schistosomiasis control programs in order to identify promptly suboptimal responders; to that effect, the WHO has issued standard procedures for control programs based on one single measurement around 3 weeks post-treatment [3]. At the same time, more work is needed to improve the current evidence-base for decision-making: in order to provide reliable information, it is important to agree on a robust statistical approach to assess drug efficacy, especially in clinical trials, and to use standardized, quality-controlled diagnostic methods that are comparable from one setting to another [18,42]. To date, both CR and ERR (the latter based on either AM or GM), are used for assessing drug efficacy. The stool and urine sampling and diagnostic approaches vary across studies, and these issues have important ramifications for drug efficacy estimates.

The controversy over the use of AMs or GMs to measure anthelmintic treatment efficacy as assessed by ERR in parasitic nematodes of cattle, and more recently in those of humans, has already been expounded in several studies, with contrasting results [43] favoring either AMs [17], or GMs (see e.g. [15]) or pointing to inadequacies of both [44]. The overall aim of this study was to compare and contrast different approaches to express treatment effects on *Schistosoma* infections in order to derive indications for standardizing future studies of drug efficacy.

The first specific objective of this paper was to compare customary measures of drug efficacy. We first considered whether efficacy assessment based on ERR changes when using AM or GM egg counts for the three predominant human *Schistosoma* species. While both means were in the same range for all species and showed a moderate level of correlation, the discrepancy between AM and GM became wider with decreasing drug efficacy. As previously reported by some [17,43,45]] but not all authors[17], GM estimates tended to be higher than AM. These findings suggest that the two means can be used interchangeably if drug efficacy is high (ERR >95%), but the difference between the two means is expected to increase as efficacy decreases. It is also worth noting that two out of the 13 studies with assessment of efficacy within 3 weeks would not meet the WHO threshold for acceptable efficacy of 90% [3] for ERR when calculated by AM, while only 8% would not meet the WHO threshold, if calculated by GM (2/24). Therefore, between the two, using AM, as suggested by WHO, appears to be more sensitive an approach to identify problems with the response to praziquantel.

We employed models to help further qualifying these findings using explanatory variables. We found that ERRs are more consistent between the AM and GM egg count values of *S*. *haematobium* and *S*. *japonicum* than of *S*. *mansoni* infection, and for school-aged children and adults than for preschool-aged children (but only when using GM). On the contrary, these findings are not accounted for by the baseline and post-treatment distributions of the raw egg counts (S2 Table), intensities of infection (S3 Table), or proportion of individuals with extreme values (S2 Table). Together, these findings are important to allow a meaningful comparison of newer studies using AM to older studies which would have used GM.

We then compared drug efficacy estimates by ERR (using AM or GM) *versus* CR. While generally used in the past, the CR is known to have some major limitations, and is no longer recommended by WHO for assessing the programmatic efficacy of drugs used in mass drug administration [12,43,45]. As expected, efficacy estimates by ERR and CR were hardly comparable, as they assess two different outcome measures (intensity *versus* presence of infection). At the onset of schistosomiasis control, the primary goal is to reduce morbidity, which is reflected by infection intensity, and hence, the ERR rather than CR might be the efficacy measure of choice. This choice is further justified by the relatively low sensitivity of widely used diagnostic methods, particularly the Kato-Katz technique for intestinal schistosomiasis [22,24,42] and the fact that current anthelmintic drugs only show low to moderate efficacies in terms of CR [13,46,47].

However, the situation is complex, and neither ERR nor CR alone provide a satisfactory description of the situation. The key questions about outcome measures are between effects on presence *versus* intensity of infection; and between measures of central tendency (for a group of individuals) *versus* individual subject responses. Our analyses indicate that the distribution of individual responses in egg excretion may be a better way of expressing results, as it comprises in one single measure drug effects on both presence and intensity of infection, and allows further detailing the distribution in centiles–which helps identifying and quantitating the presence of poor responders. ERR_AM_ and iERR are correlated, but the latter appears to be more apt to detect the presence and to quantitate the magnitude of suboptimal responses to praziquantel. More than 10% of individuals had ERR <90% in five studies, only two of which were identified by applying the ERR_AM_ 90% threshold. In these two studies, with ERR_AM_ ~82%, <30% of individuals did not achieve a 90% reduction in their egg counts, and 11–17% had no reduction at all.

These observations raise important questions as to which approach is best suited to assess drug efficacy for which purpose: while ERR is, currently, the preferred measure at the program level, identifying poor responders is important in view of tracking trends in responses and signaling potential problem areas and emergence of drug resistance. Importantly, discussions should be held with a variety of stakeholders, including drug regulatory authorities, especially if new drug applications are forthcoming.

Lastly, we compared drug efficacy estimates (ERR calculated using AM and GM, and CR) obtained by a single *versus* quadruplicate Kato-Katz thick smears for *S*. *mansoni*. Obviously, a single Kato-Katz thick smear has a lower sensitivity than multiple Kato-Katz thick smears (here ~81%) [48], and the effect and ramifications thereof for estimating drug efficacy against *Schistosoma* and other helminth infections have been discussed [42,49–51]. At the same time, collecting single stool samples and examining single Kato-Katz thick smears is operationally more feasible and less expensive than multiple sampling and multiple thick smear examinations under a microscope. We found that, when single Kato-Katz thick smears are used, the initial intensities of infection are ~2.5 times higher than with quadruplicate thick smears; efficacy estimation by ERRs based on AM and CR are ~8–9% higher, respectively, whereas ERRs based on GM are similar. Taken together, these results reflect the lower sensitivity of a single Kato-Katz thick smear, which misses low-intensity infections both on enrolment and post-treatment; the bias appears to be proportionally greater for the initial infection intensity than for the treatment outcomes. Overestimation of treatment effects may be an issue with efficacies nearing the 90% ERR threshold. In order to ascertain whether this introduces a selection bias which could affect the estimation of efficacy [52] (i.e., if the sample positive on a single Kato-Katz thick smear is different from that positive on multiple Kato-Katz thick smears), we compared both baseline FECs and efficacy estimates in these two samples using the same diagnostic technique; we found that excluding the ~19% of subjects who were negative on a single Kato-Katz selects for subjects with marginally higher initial FECs, but has ultimately no effect on efficacy estimates. Currently there is no diagnostic ‘gold’ standard method to assess *Schistosoma* response to treatment [53]. Standards may need to be tailored to the study, whether a field survey (limited by practical imperatives) or a clinical trial (which could afford more complex conditions and costly diagnosis), and whether in high or low infection intensity setting. Part of the problem, however, is the limited sensitivity of the current diagnostic methods, particularly for the detection of low intensity infections, which cannot be corrected until and unless more reliable tests become available [6,42,54].

### Conclusions

Using group means is practical when assessing sample effects, but may not be suited to detect small changes, especially those that may occur in early phases of decreasing drug efficacy. We estimate that the distribution of individual responses in *Schistosoma* egg excretion, which accounts for individual variability of responses to praziquantel treatment, allows measuring effects on both presence and intensity of infection, and helps identifying and quantitating poor responders. More research and larger databases will be required in order to identify meaningful thresholds–e.g., centile by which 90% ERR is achieved; ERR achieved by the lowest 5% or 10% centile–and also analyze in greater detail reasons for poor response.

Both approaches could be used in parallel and complement each-other. It is important to agree on standardized outcome measures that are tailored to specific purposes, such as epidemiological surveys, routine monitoring, clinical trials, morbidity control, or elimination settings. Hence, we invite other groups to contribute to this discussion and scientific inquiry.

## Supporting Information

S1 Table Results of general linear models of distinct analyses of ERR based on geometric means and ERR based on arithmetic means. (XLSX)

S2 Table Central values of the distribution of counts of eggs per gram and log transformed counts of eggs per gram measured at baseline for all studies. (XLSX)

S3 Table Frequencies and proportions of species by intensities of infection at baseline. (XLSX)
